# Sleep problems and fatigue as predictors for the onset of chronic widespread pain over a 5- and 18-year perspective

**DOI:** 10.1186/s12891-018-2310-5

**Published:** 2018-11-03

**Authors:** Katarina Aili, Maria Andersson, Ann Bremander, Emma Haglund, Ingrid Larsson, Stefan Bergman

**Affiliations:** 1 Spenshult Research and Development Center, FoU Spenshult, Bäckagårdsvägen 47, SE-302 74 Halmstad, Sweden; 2 Unit of occupational medicine, Institute of Environmental Medicine, Karolinska Institutet, Stockholm, Sweden; 3 Department of Clinical Sciences, Section of Rheumatology, Lund University, Lund, Sweden; 4 School of Business, Engineering and Science, Halmstad University, Halmstad, Sweden; 5 Department of Regional Health Research, University of Southern Denmark, Odense, Denmark; 6 Syddansk Universitet Research Unit, King Christian X Hospital for Rheumatic Diseases, Hospital of Southern Jutland, Copenhagen, Graasten Denmark; 7 School of Health and Welfare, Halmstad University, Halmstad, Sweden; 8 Primary Health Care Unit, Department of Public Health and Community Medicine, Institute of Medicine, The Sahlgrenska Academy, University of Gothenburg, Gothenburg, Sweden

**Keywords:** Musculoskeletal pain, Insomnia, CWP, Prospective study, Longitudinal study, Population study

## Abstract

**Background:**

Previous research suggests that sleep problems may be an important predictor for chronic widespread pain (CWP). With this study we investigated both sleep problems and fatigue as predictors for the onset of CWP over a 5-year and an 18-year perspective in a population free from CWP at baseline.

**Methods:**

To get a more stable classification of CWP, we used a wash-out period, including only individuals who had not reported CWP at baseline (1998) and three years prior baseline (1995). In all, data from 1249 individuals entered the analyses for the 5-year follow-up and 791 entered for the 18-year follow-up. Difficulties initiating sleep, maintaining sleep, early morning awakening, non-restorative sleep and fatigue were investigated as predictors separately and simultaneously in binary logistic regression analyses.

**Results:**

The results showed that problems with initiating sleep, maintaining sleep, early awakening and non-restorative sleep predicted the onset of CWP over a 5-year (OR 1.85 to OR 2.27) and 18-year (OR 1.54 to OR 2.25) perspective irrespective of mental health (assessed by SF-36) at baseline. Also fatigue predicted the onset of CWP over the two-time perspectives (OR 3.70 and OR 2.36 respectively) when adjusting for mental health. Overall the effect of the sleep problems and fatigue on new onset CWP (over a 5-year perspective) was somewhat attenuated when adjusting for pain at baseline but remained significant for problems with early awakening, non-restorative sleep and fatigue. Problems with maintaining sleep predicted CWP 18 years later irrespective of mental health and number of pain regions (OR 1.72). Reporting simultaneous problems with all four aspects of sleep was associated with the onset of CWP over a five-year and 18-yearperspective, irrespective of age, gender, socio economy, mental health and pain at baseline. Sleep problems and fatigue predicted the onset of CWP five years later irrespective of each other.

**Conclusion:**

Sleep problems and fatigue were both important predictors for the onset of CWP over a five-year perspective. Sleep problems was a stronger predictor in a longer time-perspective. The results highlight the importance of the assessment of sleep quality and fatigue in the clinic.

## Introduction

Experiencing musculoskeletal pain is common [1], however, the clinical presentation vary substantially.

One way of classifying musculoskeletal pain is by the number and localization of pain-sites. Localized pain is rather uncommon [2] and probably represent a different type of disorder than multisite or widespread pain. Individuals with localized pain report better general health, less pain severity and less problems with sleep than individuals with widespread pain [3]. The number of pain sites has shown to have an almost linear relationship to reduced functional ability [2] and an inverse linear relationship to general health, sleep quality and mental health [4]. If localized pain represents the one end of a pain spectra with overall better general health, chronic widespread pain (CWP) and fibromyalgia represent the other end of the spectra, having a large impact on health. Primary care patients with CWP have more comorbidities with other somatic diseases and mental illness than patients with localized chronic low back pain [5].

Population-based studies have described a CWP-prevalence of 10–17% among adult men and women [6–10]. Between 34 and 57% of those reporting CWP at baseline still reported CWP 1 to 11 years later [6–8, 10]. For early identification of patients at risk for CWP it is important to find predictors so that interventions could be directed more efficiently. Number of painful regions at baseline, age and family history of pain have been found to predict the onset of CWP [7]. Other predictors found in population-based prospective studies are somatization, illness behaviour [11, 12] and sleep disturbances [12].

Sleep problems are common among individuals reporting musculoskeletal pain. The causal sleep-pain relationship is complex and reciprocal [13, 14]. Previous studies indicate that sleep predict pain prognosis, [15–17]. The underlying mechanisms for why disturbed sleep would predict pain prognosis is not yet fully established, however there are studies indicating that sleep disturbances influence pain sensitization and pain inhibitory systems [18].

CWP, where pain is present above and below the waist, on the right and left side of the body and the axial skeleton (according to the ACR 1990 criteria for fibromyalgia [19]), is a condition commonly associated to disturbed pain systems. Prospective studies have found that sleep problems predict the onset of CWP [12, 20, 21] and resolution from CWP [17]. Sleep problems are typically assessed by (self-reported) items referring to initiating sleep, maintaining sleep and non-restorative sleep. Not feeling rested when waking up (non-restorative sleep) seems from previous studies to be the perhaps most prominent predictor out of the different types of sleep problems [17, 20]. Although this parameter ought to be closely related to fatigue, none of the studies have tried to separate the effect of fatigue from the sleep problems.

Chronic fatigue and CWP is known to common co-occur, and the disorders have been suggested to at least partly share pathogenetic pathways [22]. Sleep quality and sleep loss is related to fatigue [23, 24], but the concept of fatigue also refers to a state of mental or physical energy deprivation, rather than actual sleepiness. Although the co-occurrence of fatigue and CWP is well established, little is known about the association between fatigue and CWP prospectively.

Prospective studies of sleep and CWP share some methodological issues to handle. Musculoskeletal pain conditions, including CWP, are recurrent and regardless of using an established definition (e.g. ACR 1990 criteria for fibromyalgia), you will end up with some “border-line-cases” who move in and out of fulfilling criteria for CWP. This migration in and out of CWP has previously been reported, where almost half of those with CWP at baseline no longer reported CWP at one- or three-year follow-up [7, 8]. Although these subjects most likely have disturbances in pain regulation systems, intensity and locations of pain fluctuates, and they could falsely be categorized as “no CWP” when studied at a single time point. Studying predictors for the onset of CWP is thus complicated by that pain tend to be recurrent over time and there is a need for studies with more stable baseline classifications of individuals free from CWP at baseline.

The aim of this population-based study was to investigate if sleep problems and fatigue predicted the onset of CWP over a 5-year and up to 18- year perspective in a cohort who had not reported CWP 3 years prior to baseline.

## Method

### Population and design

This study is based on data from the EPIPAIN study, a prospective population study that was initiated in 1995 in order to investigate the prevalence and risk factors for long term musculoskeletal pain in south of Sweden. The target population for EPIPAIN was all of the 70,704 inhabitants aged 20–74, living in two municipalities and healthcare districts on the southern west coast of Sweden. For inclusion to EPIPAIN, a representative sample of subjects was selected from the official computerised population register. The register is categorised by date of birth, and the selection of subjects was made by choosing every 18th man and women respectively for each of the municipalities. A postal questionnaire was sent out in May 1995 to the 3928 selected individuals. After two postal reminders, 2425 subjects had responded to the postal survey in 1995.

Follow-up questionnaires were sent out to the subjects in 1998, 2003 and 2016. The cohort included in this study was formed out of the 1922 subjects who responded the survey in 1995 and 1998. Baseline was set to 1998 in this study with a three-year wash-out period between 1995 and 1998. Only individuals who had not reported CWP in 1995 and 1998 were included in the study. The chosen times to follow-up were 5 years (year 2003); and 18 years (year 2016).

### Chronic widespread pain

Chronic musculoskeletal pain was assessed by an overall key question: Have you experienced pain lasting more than 3 months during the last 12 months? An introduction to the question explained that the pain should be persistent or regularly recurrent in the musculoskeletal system. Pain was considered to be chronic if it had been persistent or recurrent for more than 3 months during the last 12 months.

In addition, if chronic pain was reported, the location and distribution of the pain was reported by a manikin with 18 predefined regions. Head and abdomen were not included amongst the predefined regions [7, 25].

A distinction was made between chronic regional pain (CRP) and chronic widespread pain (CWP) according to the ACR 1990 criteria for fibromyalgia [19]. According to the 1990 ACR criteria, pain was classified as widespread when present in both the left and right side of the body and also above and below the waist. In addition, axial skeletal pain (i.e. in the cervical spine, the anterior chest, the thoracic spine or the lower back) should be present. When chronic pain was present but criteria for a widespread condition were not met, the subject was classified as having CRP.

Subjects who did not report any chronic pain were labelled as “no chronic pain” (NCP).

### Sleeping problems

Problems related to sleep were assessed by four items adopted from the Uppsala Sleep Inventory (USI), which has been used in several previous epidemiological studies [26, 27]: How much of a problem do you have with: (1) Falling asleep at night? (2) Waking up during the night? (3) Waking up too early in the morning? (4) Not feeling rested after sleep?

The problems were recorded on a five-point Likert scale: [1]=no problems, [2]=minor problems, [3]=moderate problems, [4]=severe problems, and [5]=very severe problems. Those who had responded “moderate problems”, “severe problems” or “very severe problems” were considered to have sleep problems.

The sleep problems were treated as four separate variables of sleep, 1) Difficulties initiating sleep; 2) Difficulties maintaining sleep; 3) Early morning awakening; and 4) Non-restorative sleep.

Fatigue was estimated by the vitality subscale from the SF-36 questionnaire [28, 29]. The items from the subscale assess how great part of the time the last 4 weeks one have felt 1) alert and strong; 2) full of energy; 3) worn out; 4) tired. The response rates range from [1] “All the Time” to [6] “None of the time”. The scale was converted according to the SF-36 manual into a scale ranging from 0 to 100, were a higher score indicate less problem with fatigue. Three levels of fatigue (low, intermediate, high) was constructed by dividing the included cohort’s scoring into tertiles. “Low fatigue” then represented scorings of 85–100; “intermediate fatigue” represent scorings of 70–84 and “high fatigue” represent scorings between 0 and 69. The vitality subscale from SF-36 has been widely used in studies of populations with musculoskeletal pain, including fibromyalgia, and is a validated measure of fatigue [30].

### Potential confounders

Mental health [20, 31] and socio-economy [21] as well as baseline pain [20] has previously been found to be associated to both sleep problems and CWP. The potential confounders considered in this study were age, gender, number of musculoskeletal pain sites, socio economy and mental health at baseline.

*Number of (chronic) musculoskeletal pain sites* were reported by a manikin, as described above, with eighteen possible regions.

*Mental Health* was assessed by the subscale MH from SF-36, including five items referring to the last 4 weeks: How much of the time during the last 4 weeks have you… 1) been a very nervous person?; 2) felt so down in the dumps that nothing could cheer you up?; 3) felt calm and peaceful; 4) felt downhearted and blue?; 5) been a happy person? Three levels of mental health (poor, intermediate, good) was constructed by dividing the included cohort’s scoring into tertiles. “good mental health” then represent scores between 93 and 100; “intermediate mental health” scores between 84 and 92; and “poor mental health” scores between 0 and 83.

*Socio economy* as measured by Socio economic groups according to Statistics Sweden was based on self-reported occupation in 1995. Four groups of socio-economic classes were formed; Manual workers, assistant non-manual employees, intermediate/higher non-manual employees and others (including self-employed, farmers, housewives, students).

### Ethics

All study participants signed an informed consent before entering the study. The study was approved by the Regional Ethical Review Board, Faculty of Medicine, University of Lund, Sweden (Dnr LU 389–94; Dnr 2016/132).

### Statistics

The included cohort and the subjects that had been excluded due to reporting CWP in 1995 and/or 1998 were compared with respect to age, gender, the four sleep parameters, fatigue, number of pain regions, mental health and socio economy (based on type of occupation) at baseline. The prevalence of exposed to each sleep problem and fatigue respectively within the different categories of gender, pain regions, mental health and socio economy were compared. Pearson’s chi-square test and independent t-test were used in the analyses for difference.

Odds ratios for CWP were calculated using binary logistic regression analysis. The effect of the four different sleep problems and fatigue were tested separately, for each year of follow-up.

Firstly, the four different sleep parameters and fatigue were tested in separate models in order to see if the different sleep problems or fatigue were of different importance. The predictors and the potential confounders were tested in binary logistic regression analysis, adjusting for age and gender. Multivariate analyses were then performed. Due to the complex relationship between pain, sleep and mental ill health [13], the analyses were presented in three models: 1) adjusting for age, gender, socio economy and mental health and 2) adjusting for age, gender socio economy and number of pain sites; and 3) adjusting for age, gender, socio economy, mental health and number of pain sites. The decision to let either number of pain sites or mental health enter models 1 and 2 and to let them act together in model 3 was made based on an attempt to be transparent in how the two affect the results, separately and together.

Secondly, the independent effect of sleep and fatigue on CWP was tested, and the two variables were included in the same model. To get one sleep variable instead of four separate sleep variables, sleep problems were categorized into having 0–4 of the (4) sleep problems. Again, number of pain sites and mental health were included in different models, and simultaneously. The effect of number of sleep problems, mental health and socio-economy were analysed in a “Model A”, including also age and gender. In separate analyses Model A was tested when including fatigue, and number of pain sites.

All logistic regression analyses were performed twice, one for CWP at the five-year follow-up, and once for CWP at the 18-year follow-up.

The analyses were performed with the SPSS 21 Statistical Package.

## Results

In all, 1922 subjects responded to the survey in 1995 and 1998. Missing data on pain questions were found in 49 cases in 1995, and 22 cases in 1998. In all, 1852 subjects had data from both 1995 and 1998 and could thus enter the analysis. Among these, 340 individuals had reported CWP in either 1995 or 1998 and were excluded from the analysis.

The excluded subjects were significantly older, were more likely to be female, to have problems with sleep and differed in socio economy from the included individuals. Further, the excluded individuals generally rated higher fatigue, poorer mental health and more pain severity. See Table 1.

**Table 1:** Presenting baseline characteristics of the cohort with baseline stable NCP or CRP and individuals excluded from the study due to reporting CWP in − 95 and/or − 98. Prevalence of baseline exposures and covariates in included and excluded subjects, and history of CWP in excluded individuals. Significance of differences between included and excluded subjects are presented as *p*-values (tested with t-test and chi^2^-test respectively)

	Included cohort (stable NCP or CRP) (*n* = 1512)	Excluded subjects (CWP in 1995 and/or 1998) (*n* = 340)	*p*-value for diff. Incl. vs excl.
Age (1998)			< 0.01
Mean (sd)	49 (15)	56 (13)	
Gender; n (%)			<.01
Male	729 (48)	111 (33)	
Female	783 (52)	229 (67)	
Initiating sleep; n (%)			<.01
No Problem	1211 (81)	175 (53)	
Problem	282 (19)	158 (47)	
Maintaining sleep; n (%)			<.01
No Problem	1024 (69)	109 (33)	
Problem	461 (31)	220 (67)	
Early awakening; n (%)			<.01
No Problem	1149 (78)	145 (44)	
Problem	331 (22)	186 (56)	
Non restorative sleep; n (%)			<.01
No Problem	1075 (73)	123 (37)	
Problem	404 (27)	207 (63)	
Fatigue^a^			<.01
Mean (sd)	72 (21)	44 (25)	
Number of Regions			<.01
0	1096 (73)	42 (12)	
1–2	204 (13)	10 (3)	
3–5	177 (12)	80 (24)	
6–11	35 (2)	152 (45)	
12–18	0	56 (17)	
Mental Health^b^			< .01
Mean (sd)	84 (17)	69 (23)	
Socio economy^c^			< .01
High non manual work	440 (29)	48 (14)	
Ass non manual	212 (14)	55 (16)	
Manual	664 (44)	196 (58)	
Others	196 (13)	41 (13)	
CWP history at baseline			
CWP in −95 only	–	103 (30)	
CWP in −98 only	–	101 (30)	
CWP in both −95 and − 98	–	136 (40)	

^a^Assessed by SF-36 vitality scale; ^b^Assessed by SF-36 Mental health scale; ^c^Classified by occupation – intermediate/higher non-manual employees, Assistant non-manual employees, Manual workers and “others”

**Fig. 1:**
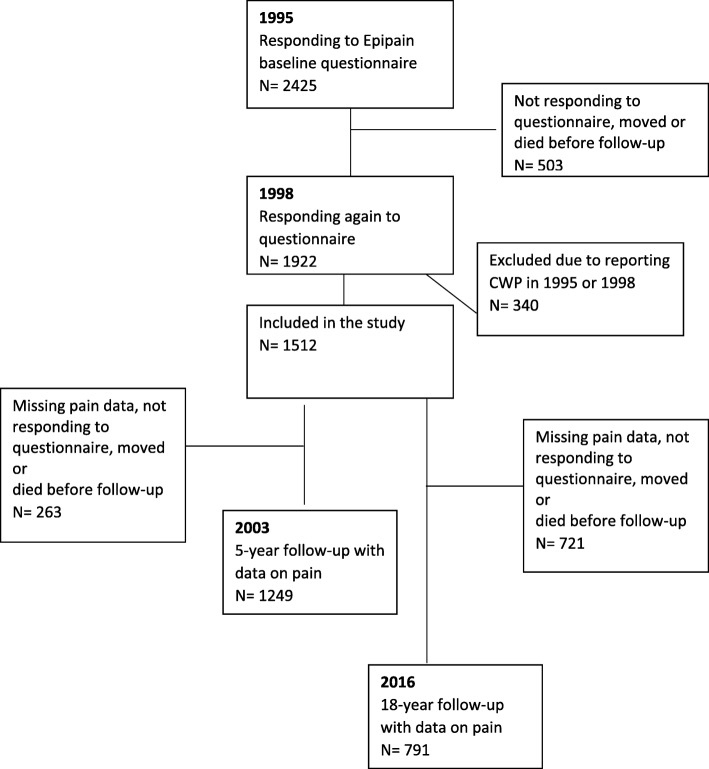
Flow-chart showing participation in the study

Out of the 1512 individuals who fulfilled the inclusion criteria, 1249 had responded to the pain items in 2003, and were thus eligible for the analysis referring to the 5-year follow up; and 791 responded to the items in 2016 and were thus eligible for the analysis referring to the 18-year follow-up. See flow-chart in Fig. 1.

Out of the included subjects, 89 (7%) had CWP in the 5-year follow-up and 103 (13%) had CWP in the 18-year follow-up. There was no difference in proportion of men and women reporting CWP in the 5-year follow up (8% vs 6%, *p* = 0.14). However, there were significantly larger proportion of females than men reporting CWP in the 18-year follow-up (16% vs 10%, *p* = 0.01). There was a significantly larger proportion who reported CWP 5- and 18 years later among those with sleeping problems and fatigue compared to those without sleeping problems and fatigue at baseline. See Table 2. The same was seen for differences in number of pain regions and level of mental health. Data not shown.

**Table 2:** Presenting prevalence of baseline sleep problems and fatigue. Cross tabulation of baseline sleep problems and CWP at respective follow-up. Differences between no CWP and CWP cases in respective year for follow-up have been tested with chi^2^-test

	5-year follow-up(*N* = 1249)			18-year follow-up (*N* = 791)		
	No CWP	CWP	*p*	No CWP	CWP	*p*
	*N* = 1160	*N* = 89		*N* = 688	*N* = 103	
Initiating sleep; n (%)			< 0.01			< 0.01
No Problem	950 (95)	53 (5)		580 (89)	70 (11)	
Problem	198 (85)	34 (15)		103 (76)	32 (24)	
Maintaining sleep; n (%)			< 0.01			< 0.01
No Problem	811 (95)	41 (5)		500 (91)	52 (9)	
Problem	330 (88)	46 (12)		183 (78)	50 (22)	
Early awakening; n (%)			< 0.01			< 0.01
No Problem	909 (95)	49 (5)		556 (89)	70 (11)	
Problem	228 (86)	37 (14)		124 (80)	32 (20)	
Non restorative sleep; n (%)			< 0.01			< 0.01
No Problem	856 (95)	42 (5)		525 (90)	56 (10)	
Problem	283 (87)	44 (13)		159 (78)	45 (22)	
Fatigue; n (%)			< 0.01			< 0.01
Low	455 (97)	14 (3)		285 (91)	27 (9)	
Intermediate	355 (94)	21 (6)		230 (90)	26 (10)	
High	346 (87)	53 (13)		173 (78)	50 (22)	

One fourth of those who reported all four sleep problems (concurrently) at baseline had CWP 5 years later, and one third had CWP 18 years later. Among those reported none of the sleeping problems at baseline, 4% reported CWP 5 years later and 8% 18 years later.

In all, 769 individuals had data on pain at all three time points. Looking at changes in pain between the 5-year follow-up and the 18-year follow-up, 59% stayed in the same pain group (NCP, CRP or CWP). Out of the 52 who reported CWP in the 5-year follow-up (and who had data also at the 18-year follow-up), 56% (*n* = 29) reported CWP also in the 18-year follow-up. In all, 100 participants reported CWP in the 18-year follow-up. See Fig. 1.

### Exposed at baseline

**Fig. 2:**
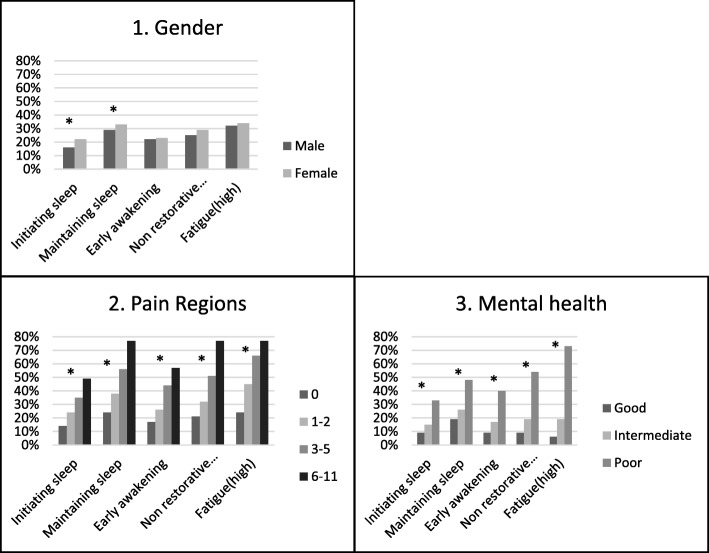
Presenting baseline data. The figure presents the percentage within each category of 1) Gender; 2) Number of pain regions and 3) Mental health, reporting problems with: Initiating sleep, Maintaining sleep, Early awakening, Non-restorative sleep, and Fatigue respectively. *p for difference < 0.05

As presented in Fig. 2, there was a significantly higher proportion of females reporting problems with initiating sleep and problems with maintaining sleep at baseline. No gender differences were seen for problems with early awakening, non-restorative sleep or fatigue.

**Fig. 3:**
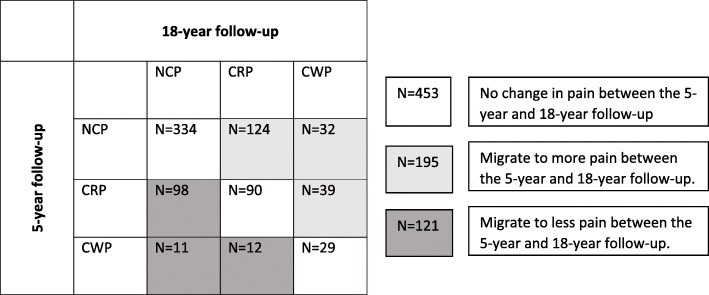
Describing migration between the pain groups over time. The figure describe migration between reporting No Chronic Pain (NCP); Chronic Regional Pain (CRP) and Chronic Widespread Pain (CWP) between the 5-year follow-up and 18-year follow-up. Including the (*N* = 769) with data on pain in both 5-year follow-up and 18-year follow-up

There were a larger proportion among individuals reporting more number of pain regions who reported problems with sleeping (significant for all sleep parameters) and fatigue. The same trend was seen for mental health, where subjects reporting poor mental health were more likely to also report problems with sleep (all sleep parameters) and fatigue.

### Predictors for CWP in crude analysis

After adjusting for age and gender, all sleep parameters, fatigue (SF-36 Vitality), number of pain regions and mental health (SF-36) predicted the onset of new CWP in the 5-year- and 18-year follow-up. Being a manual worker in 1995 predicted the onset of new CWP in the 5-year follow-up. See Table 3.

**Table 3:** Presenting results from univariate model (adjusted for age and gender). Odds ratio (OR), 95% confidence intervals (95% CI) and p-value (p) for reporting CWP in the 5-year and 18-year follow-up respectively. Number of individuals per category of exposure at baseline are presented as N for the 5-year follow-up and 18-year follow-up respectively

	N	CWP 5-year OR (95% CI)	*p*	N	CWP 18-year OR (95% CI)	*p*
Gender						
Male	586	1		345	1	
Female	663	1.44 (0.92–2.24)	.107	446	1.77 (1.14–2.75)	.011
Initiating sleep^a^						
No problem	1003	1		650	1	
Problem	232	2.82 (1.77–4.50)	< .001	135	2.49 (1.55–4.00)	< .001
Maintaining sleep^a^						
No problem	852	1		552	1	
Problem	376	2.53 (1.61–3.98)	< .001	233	2.74 (1.75–4.28)	< .001
Early awakening^a^						
No problem	958	1		626	1	
Problem	265	2.83 (1.79–4.46)	< .001	156	1.97 (1.23–3.15)	.005
Non-restorative^a^						
No problem	898	1		581	1	
Problem	327	3.15 (2.02–4.92)	< .001	204	2.62 (1.70–4.04)	< .001
Fatigue^b^						
Low	469	1		312	1	
Intermediate	376	1.99 (1.00–3.99)	.051	256	1.20 (0.68–2.11)	.533
High	399	4.93 (2.69–9.05)	< .001	223	2.96 (1.78–4.92)	< .001
Pain regions						
0	904	1		571	1	
1–2	167	1.61 (0.78–3.32)	.202	113	3.03 (1.72–5.31)	< .001
3–5	146	7.40 (4.40–12.42)	< .001	89	5.33 (3.05–9.31)	< .001
6–11	32	12.40 (5.50–28.02)	< .001	18	13.38 (4.99–35.89)	< .001
Mental health^c^						
Good	365	1		234	1	
Intermediate	487	1.89 (0.96–3.73)	.065	326	1.37 (0.77–2.45)	.288
Poor	388	3.87 (2.05–7.34)	<.001	229	2.70 (1.53–4.75)	.001
Socio economy^d^						
High non manual	384	1		285	1	
Ass non manual	178	1.98 (0.93–5.21)	.076	107	1.17 (0.59–2.96)	.660
Manual	537	2.74 (1.51–4.94)	.001	321	1.45 (0.89–2.36)	.136
Others	150	1.45 (0.61–3.55)	.395	78	1.25 (0.58–2.68)	.574

^a^*Problem* = [Moderate problems - very severe problems]; *No Problems* = [no problems - minor problems]. ^b^ Based on scorings on SF-36 vitality scale [Low = 85–100; Intermediate = 70–84; High = 0–69]

^c^Based on scorings on SF-36 Mental Health scale [Good = 93–100; Intermediate = 84–92; Poor = 0–83]; ^d^ Classified by occupation – intermediate/higher non-manual employees, Assistant non-manual employees, Manual workers and “others”

### The effect of sleep as a predictor for CWP in multivariate models

Over a five-year perspective, all sleep parameters and fatigue predicted the onset of CWP, irrespective of age, gender, mental health and socio-economy (model 1). When adjusting for number of pain sites at baseline instead of mental health (model 2), fatigue and all sleep parameters except maintaining sleep predicted the onset of CWP. Finally, adjusting for age, gender, socio-economy, mental health and number of pain sites (model 3), none of the sleep parameters or fatigue significantly predicted the onset of CWP 5 years later.

Over the 18-year perspective, fatigue and all sleep parameters except early awakening significantly predicted CWP when adjusting for age, gender, socio-economy and mental health. However, when including number of pain sites at baseline in the model separately (model 2) and simultaneously with mental health (model 3), maintaining sleep was the only parameter that remained significant in the 18-year follow-up.

Results are presented in Table 4.

**Table 4:** Results from multivariate analysis. Presenting the effect of difficulties initiating sleep, difficulties maintaining sleep, early morning awakening, non-restorative sleep and fatigue on the odds ratio (OR) for reporting CWP in the 5-year and 18-year follow-up respectively. The OR, 95% confidence intervals (95% CI) and p-value (p) are presented in model 1–3. *N* = 1249 entered the 5-year follow-up analyses; *N* = 791 entered in the 18-year follow-up analyses

	Adjusted for age gender, mental health^c^, socio-economy^d^		Adjusted for age, gender, socio-economy^d^and number of pain sites		Adjusted for age, gender, socio-economy^d^, mental health^c^ and number of pain sites	
	CWP 5-year	CWP 18-year	CWP 5-year	CWP 18-year	CWP 5-year	CWP 18-year
	OR (95% CI)	OR (95% CI)	OR (95% CI)	OR (95% CI)	OR (95% CI)	OR (95% CI)
Initiating sleep^a^						
No Problem	1	1	1	1	1	1
Problem	1.91 (1.16–3.14)*	1.93 (1.18–3.18)*	1.71 (1.03–2.85)*	1.68 (1.00–2.81)	1.37 (0.81–2.34)	1.51 (0.89–2.57)
Maintaining Sleep^a^						
No problem	1	1	1		1	1
Problem	1.85 (1.14–3.01)*	2.25 (1.40–3.61)*	1.60 (0.98–2.63)	1.88 (1.16–3.05)*	1.32 (0.79-2.21)	1.72 (1.05–2.84)*
Early awakening^a^						
No problem	1	1			1	
Problem	2.00 (1.23–3.27)*	1.54 (0.94–2.54)	1.71 (1.04–2.83)*	1.32 (0.79–2.21)	1.45 (0.86–2.43)	1.18 (0.69–2.01)
Non-restorative^a^						
No problem	1	1			1	1
Problem	2.27 (1.37–3.75)*	2.04 (1.26–3.29)*	1.90 (1.15–3.13)*	1.57 (0.97–2.56)	1.51 (0.88–2.58)	1.39 (0.83–2.34)
Fatigue^b^						
Low	1	1	1	1	1	1
Intermediate	1.75 (0.83–3.66)	1.09 (0.60–2.00)	1.71 (0.84–3.45)	1.05 (0.59–1.89)	1.48 (0.70–3.15)	0.97 (0.52–1.79)
High	3.70 (1.76–7.84)*	2.36 (1.24–4.50)*	2.59 (1.34–3.47)*	1.62 (0.92–2.85)	1.93 (0.87–4.26)	1.34 (0.69–2.68)

^a^*Problems* = [Moderate problems - very severe problems]; *No Problems* = [no problems - minor problems]. ^b^ Based on scorings on SF-36 vitality scale [Low = 85–100; Intermediate = 70–84; High = 0–69]; ^c^Based on scorings on SF-36 Mental Health scale [Good = 93–100; Intermediate = 84–92; Poor = 0–83]; ^d^Classified by occupation – intermediate/higher non-manual employees, Assistant non-manual employees, Manual workers and “others”

*p < 0.05

In all, 785 individuals did not report any of the sleeping problems at baseline (fatigue not included), 268 reported one of the problems, 167 two, 128 three and 117 subjects reported to have all four sleep problems.

In a multivariate model (Model A) including age, gender, mental health, socio-economy and number of sleep problems, reporting four sleep problems, reporting poor mental health and the socio-economy-parameter manual work were all associated with the onset of CWP 5 years later. Adding fatigue to the model showed that sleep problems, fatigue and manual work predicted CWP independently from each other, however mental health did not remain significant in the model. When, instead, including number of pain sites to Model A, reporting four sleep problems, reporting at least three pain-sites and manual work predicted the onset of CWP. Reporting at least three pain sites at baseline and manual work were the only parameters that remained significant when adding also fatigue to the model. See Table 5.

**Table 5:** Presenting odds ratios (OR) and 95% confidence intervals (95% CI) for CWP 5 years later. Model A includes age, gender, mental health, socio economy and number of sleep problems

	Model A	Model A + fatigue	Model A + number of pain regions	Model A + number of pain regions and fatigue
	OR (95% CI)	OR (95% CI)	OR (95% CI)	OR (95% CI)
No of sleep problems				
0	1	1	1	1
1	1.13 (0.57–2.27)	1.00 (0.50–2.03)	0.93 (0.45–1.89)	0.88 (0.43–1.80)
2	1.01 (0.44–2.32)	0.84 (0.36–1.96)	0.73 (0.31–1.72)	0.68 (0.29–1.62)
3	1.71 (0.77–3.81)	1.39 (0.62–3.15)	1.14 (0.49–2.63)	1.05 (0.45–2.45)
4	4.00 (2.03–7.91)**	3.06 (1.51–6.21)**	2.18 (1.04–4.56)*	1.99 (0.93–4.24)
Fatigue^a^				
Low		1		1
Intermediate		1.62 (0.76–3.45)		1.47 (0.69–3.16)
High		2.68 (1.21–5.96)*		1.59 (0.68–3.71)
Pain regions at baseline				
0			1	1
1–2			1.39 (0.64–3.02)	1.34 (0.62–2.91)
3–5			4.89 (2.73–8.76)**	4.53 (2.47–8.30)**
> 6			8.24 (3.37–20.15)**	7.78 (3.13–19.26)**
Mental health^b^				
Good	1	1	1	1
Intermediate	1.60 (0.79–3.23)	1.26 (0.60–2.66)	1.42 (0.70–2.90)	1.23 (0.58–2.62)
Poor	2.41 (1.17–4.93)*	1.42 (0.62–3.24)	1.93 (0.93–4.02)	1.54 (0.67–3.56)
Socio economy (work)^c^				
High non-manual	1	1	1	1
Ass non-manual	1.69 (0.75–3.82)	1.69 (0.75–3.84)	1.87 (0.81–4.32)	1.86 (0.80–4.28)
Manual	2.75 (1.47–5.13)**	2.73 (1.46–5.12)**	2.34 (1.23–4.44)*	2.34 (1.23–4.46)*
Other	1.40 (0.56–3.49)	1.35 (0.54–3.39)	1.27 (0.50–3.27)	1.26 (0.49–3.23)

^a^Based on scorings on SF-36 vitality scale [Low = 85–100; Intermediate = 70–84; High = 0–69];^b^Based on scorings on SF-36 Mental Health scale [Good = 93–100; Intermediate = 84–92; Poor = 0–83]; ^c^Classified by occupation – intermediate/higher non-manual employees, Assistant non-manual employees, Manual workers and “others”

*p < 0.05; **p < 0.01

Over an 18-year perspective, sleep problems were stronger independent predictor for CWP. Out of the included parameters, only sleep problems and number of pain regions at baseline significantly and independently, predicted CWP. See Table 6.

**Table 6:** Presenting odds ratios (OR) and 95% confidence intervals (95% CI) for CWP 18 years later. Model A includes age, gender, mental health, socio economy and number of sleep problems

	Model A	Model A + fatigue	Model A + number of pain regions	Model A + number of pain regions and fatigue
	OR (95% CI)	OR (95% CI)	OR (95% CI)	OR (95% CI)
No of sleep problems				
0	1	1	1	1
1	1.82 (1.00–3.34)	1.73 (0.93–3.20)	1.57 (0.84–2.95)	1.57 (0.83–2.96)
2	1.38 (0.65–2.91)	1.27 (0.59–2.71)	1.03 (0.48–2.22)	1.02 (0.47–2.23)
3	2.44 (1.19–5.00)*	2.17 (1.03–4.56)*	1.78 (0.84–3.80)	1.75 (0.81–3.82)
4	3.95 (1.90–8.20)**	3.33 (1.55–7.13)**	2.36 (1.06–5.23)*	2.29 (1.01–5.18)*
Fatigue^a^				
Low		1		1
Intermediate		0.96 (0.51–1.79)		0.89 (0.47–1.67)
High		1.79 (0.89–3.60)		1.13 (0.54–2.37)
Pain regions at baseline				
0			1	1
1–2			2.83 (1.57–5.12)**	2.76 (1.52–5.02)**
3–5			4.03 (2.20–7.37)**	3.84 (2.05–7.20)**
> 6			8.63 (3.05–24.43)**	8.24 (2.88–23.63)**
Mental health^b^				
Good	1	1	1	1
Intermediate	1.22 (0.67–2.25)	1.15 (0.61–2.18)	1.21 (0.65–2.24)	1.21 (0.63–2.31)
Poor	1.75 (0.92–3.13)	1.25 (0.59–2.66)	1.36 (0.70–2.63)	1.27 (0.59–2.72)
Socio economy (work)^c^				
High non-manual	1	1	1	1
Ass non-manual	1.22 (0.61–2.47)	1.25 (0.62–2.54)	1.21 (0.58–2.49)	1.22 (0.59–2.53)
Manual	1.49 (0.90–2.48)	1.50 (0.90–2.51)	1.40 (0.83–2.38)	1.41 (0.83–2.38)
other	1.24 (0.56–2.77)	1.26 (0.56–2.83)	1.29 (0.56–3.00)	1.31 (0.56–3.04)

^a^Based on scorings on SF-36 vitality scale [Low = 85–100; Intermediate = 70–84; High = 0–69]

^b^Based on scorings on SF-36 Mental Health scale [Good = 93–100; Intermediate = 84–92; Poor = 0–83]; ^c^Classified by occupation – intermediate/higher non-manual employees, Assistant non-manual employees, Manual workers and “others”

*p < 0.05; **p < 0.01

## Discussion

The aim of this study was to investigate if sleep problems and fatigue predict the onset of CWP five- and 18 years later. The results from this study indicate that in a cohort free from CWP at baseline, difficulties initiating sleep, maintaining sleep, early morning awakening, non-restorative sleep and fatigue respectively predicted the onset of CWP in a 5-year perspective irrespective of age, gender, socio-economy and mental health. This was true also over an 18-year perspective, except for the sleep parameter early morning awakening. When adding number of pain sites in the model none of the sleep parameters or fatigue predicted the onset of CWP in the 5-year follow-up. However, problems with maintaining sleep consistently predicted the onset of CWP 18 years later in all models.

When adding all sleep problems together, receiving a parameter measuring 0–4 sleep problems, sleep problems predicted the onset of CWP 5 years later irrespective of age, gender, mental health and socio-economy. Further, sleep problems and fatigue predicted the onset of CWP 5 years later irrespective of each other. However, in a full model including the parameters mentioned above plus sleep problems, fatigue and number of pain sites, only ≥3 pain sites at baseline and having manual work at baseline significantly predicted the onset of CWP 5 years later. Over an 18-year perspective however, reporting 4 sleep problems at baseline and reporting pain at baseline (at least 1–2 pain sites) were the only predictors that remained significant when included in a full multivariate model.

The over-all results, that sleep is a predictor for CWP, is in line with what previous studies have found [12, 21, 31, 32]. These studies have used follow-ups of 15 months, 6 years and 11 years. Our findings, that sleep seems to be an important predictor also over very long time-spans are supported by prospective studies showing that insomnia predict chronic widespread musculoskeletal pain 11 years later [32]. Although the previous studies have used a study design where following a cohort free from CWP at baseline, this study is the first study being able to exclude also those who had had CWP 3 years prior to baseline.

The mechanistic relationship between sleep problems and chronic pain is not yet clear. One suggested mechanism is that insufficient sleep alters the processes of pain habituation and sensitization, and increase vulnerability to chronic pain [18]. The purpose of using a wash-out period was to distinguish between regional pain (presumably without central sensitization) and CWP (where disturbed pain systems are common). We then found that over a five-year perspective, the sleep-CWP relationship was not explained by pain regions at baseline or mental health. Further, fatigue predicted the onset of CWP independently from mental health and sleep problems, but not independently from number of pain regions at baseline. This implies that the association between fatigue and CWP is explained by number of pain sites rather than mental health and sleep problems. In the longer time-perspective of 18 years however, fatigue did not predict the onset of CWP independently from sleep problems. Possibly, this could be interpreted as fatigue being more associated with disturbances in pain systems, whereas sleep problems not only are associated with disturbed pain systems, but also indicates a vulnerability to chronic pain, preceding disturbances of the system.

Using the wash-out period, allowing only those who had not reported CWP both at baseline and 3 years prior to baseline, we attended to capture *new* onset CWP. This is however more likely to be true for the results found in the 5-year follow-up than the 18-year follow-up. One of the reasons for why sleep problems predict CWP also over longer time periods may be that sleep problems [6] and fatigue [8] predict persistence of CWP. At the 18-year follow-up it is reasonable to believe that what we see is the combined effect of sleep problems on the onset of CWP, as well as persistence of CWP. As shown in Fig. 3, a large part of the study participants stayed in the same pain group between the 5-year and 18-year follow-up. However, 44% of those reporting CWP in the 5-year follow-up reported “No Chronic Pain” or “Chronic Regional Pain” in the 18-year follow-up and the mere part of those reporting CWP in the 18-year follow-up (70%) did not report CWP in the 5-year follow-up. We have not found any previous prospective studies investigating the effect of predictors for CWP both over a “shorter” time span (5 years) and a very long time-span (18 years) simultaneously.

In this study we found that difficulties maintaining sleep, and possibly difficulties initiating sleep, seems to be stronger independent predictors for CWP in a long-term perspective (18 years) than in a 5-year perspective. The opposite trend was seen for early morning awakening, non-restorative sleep and fatigue. Other studies of sleep and CWP that has specified single component of sleep in their analysis [17, 20] point out non-restorative sleep and trouble initiating sleep as the two important components, when investigating the onset of CWP 3 years later (among older adults) [20] and resolution from CWP 15 months later [17]. A previous study [31] suggest that difficulties initiating or maintaining sleep, could worsen a person’s depressive symptoms over time, which may explain some of our findings. A recent review concludes that deterioration in sleep is associated with an increased risk for developing a pain condition [33]. Future studies should investigate the variance of both sleep and CWP over time for better understanding of the clinical meaning of the findings in the present study.

We chose to include mental health and number of pain regions in separate models in the analysis. Pain, sleep problems and mental health are known to commonly co-occur. It was not under the scope of this study to unravel the different pathways through which sleep, pain, fatigue and mental health interact. However, knowing that the relationship is complex, we wanted to be as transparent as possible when exploring the effect of sleep and fatigue on CWP. A recent review [13] investigating the relationship between sleep and pain suggest that sleep, pain and negative mood share variance after presenting studies that suggest sleep to be a mediator between the relationship between pain and depression [34]; pain to mediate the relationship between sleep and depression [35]; and negative mood to mediate the relationship between sleep and pain [36]. Sleep problems have further been found to predict the development of depression among individuals with persistent pain [37]. Another recent study found that insomnia and sleep duration are risk factors for developing chronic multisite pain and that the mediation effect of depressive symptoms, at least partially explained the increased risk [31]. Further, CWP and fatigue commonly co-exist, and it has been shown that many of those reporting both fatigue and CWP also report anxiety or depression [38].

Having had pain previously is an important predictor for reporting more pain sites at follow-up [39]. In this study, individuals who had reported CWP 3 years prior to baseline were excluded from entering the analyses to get a more stable baseline classification of “no CWP”. Most other studies do not include a wash-out period when studying the onset of CWP. As shown in Tables 1, 30% of the excluded reported CWP in 1995, but not in 1998, which means that if we had chosen to not use a wash-out period, we would have classified them as “no CWP” at baseline (− 98). The included cohort differed significantly from the excluded group in all parameters of interest in this study; age, gender, the four sleep parameters, fatigue, number of pain regions, pain severity, mental health and socio economy and the excluded group were with respect to these parameters supposedly a more vulnerable group of individuals in general. These differences strengthen our hypothesis that these factors are of importance. When interpreting the result, it is important to bear in mind that they are based on a cohort that due to the wash-out period include a higher proportion of individuals who are supposedly less vulnerable to CWP than a random sample from a general population would be.

Previous population-based studies have established a higher CWP-prevalence among women than men [11, 40]. Unexpectedly, in this study, female gender predicted the onset of CWP in the 18-year follow-up, but not in the 5-year follow-up. One explanation for this may be the use of a wash-out period for CWP, as discussed above. The effect of gender that was seen in the 18-year follow-up may reflect that females have higher risk for persistent CWP than men. A previous study on the cohort [7] found that women had higher risk for developing CWP, and higher risk (however non-significant) than men for persistent CWP. The gender difference in risk for long-term persistence of CWP has been seen also in other cohorts [6]. However, a British study with one-year follow-up did not find any gender difference in persistence of CWP [8].

There are some methodological issues to be considered when interpreting the results from this study. The results rely upon self-reported data from questionnaires. Individuals classified as CWP in this study may have differed if they were diagnosed by a physician. By the criterion used in this study, subjects could have reported up to 11 sites without fulfilling the criteria for CWP. One could fulfil the CWP criteria if reporting pain from only three sites, thus the individuals classified as CWP may be heterogeneous. This approach for classifying CWP [19] is however the most commonly used in population-studies, and our results are therefore comparable to other studies [6, 8–12, 17, 20, 21, 40].

The parameter indicating socio economy may have some problems. The classification was made based on occupation according to a classification system by Statistics Sweden, 1982. In 1998, when data was assessed from which the socio-economy parameter was classified in this study, this classification was considered accurate to use. We chose to use this parameter in this study to get an idea of the impact of socio economy and/or type of work, although we are aware it is not a perfect estimation.

Another issue worth mentioning is the repeated analysis, due to our decision to perform separate analysis in models including mental health and number of pain sites, separately and simultaneously. The multiple analysis increases the risk for false positive associations. In the age-gender adjusted analysis (presented as crude analysis) of the predictors, the *p*-values were very low (below 0.001). This imply that there is a very small risk for false positive results in the “crude” relationship between the investigated predictors and CWP. However, as moving on and analysing the predictors in several models, with less convincing p-values, the risk for false positive results increase. This is a limitation with the study.

Another issue is the potential nonparticipation bias. In the five-year follow-up, the response rate was 90% and in the 18-year follow-up, the response rate was 63%. Especially the response rate of the latter follow-up could potentially have caused bias. However, in comparison to other studies with this long-term follow-up, the response rate is within a range one would expect.

## Conclusions

This prospective populations study showed that all the investigated sleep problems, (initiating sleep, maintaining sleep, early awakening and non-restorative sleep) as well as fatigue are important predictors for the future development of CWP both over a 5-year, and 18-year perspective. Problems with maintaining sleep was a weaker predictor in the five-year follow-up but was the only sleep parameter that predicted the onset of CWP over an 18-year perspective irrespective of both mental health and number of pain sites.

Reporting all four sleep problems simultaneously was a strong predictor for CWP 5 years- and 18-years later. Fatigue predicted the onset of CWP 5 years later irrespective of sleep problems, age, gender, socio-economy and mental health, but not independently from pain sites. The results from this study are in line with what previous prospective studies have shown, and with this study we add knowledge of the importance of fatigue as predictor over a shorter perspective. This highlights the importance of the assessment of sleep quality and fatigue in the clinic. This study suggests that the fatigue-CWP association is explained by number of pain sites (and possibly disturbed pain systems) rather than mental health or sleep problems. This study also suggests that sleep problems may indicate a vulnerability to chronic pain at an earlier stage.

## Abbreviations

CI: Confidence interval
CRP: Chronic regional pain
CWP: Chronic widespread pain
NCP: No chronic pain
